# Blood-brain barrier permeability and physical exercise

**DOI:** 10.1186/s12974-019-1403-x

**Published:** 2019-01-24

**Authors:** Marta A. Małkiewicz, Arkadiusz Szarmach, Agnieszka Sabisz, Wiesław J. Cubała, Edyta Szurowska, Paweł J. Winklewski

**Affiliations:** 1 Department of Human Physiology, Faculty of Health Sciences, Medical University of Gdansk, Tuwima Str. 15, 80-210 Gdansk, Poland; 2 Department of Psychiatry, Faculty of Medicine, Medical University of Gdansk, Gdansk, Poland; 3 2-nd Department of Radiology, Faculty of Health Sciences, Medical University of Gdansk, Gdansk, Poland; 4 Department of Clinical Anatomy and Physiology, Faculty of Health Sciences, Pomeranian University of Slupsk, Slupsk, Poland

**Keywords:** Blood-brain barrier permeability, Physical exercise, Inflammation, Brain renin-angiotensin system, Central autonomic function, Kynurenine pathway

## Abstract

In this narrative review, a theoretical framework on the crosstalk between physical exercise and blood-brain barrier (BBB) permeability is presented. We discuss the influence of physical activity on the factors affecting BBB permeability such as systemic inflammation, the brain renin-angiotensin and noradrenergic systems, central autonomic function and the kynurenine pathway. The positive role of exercise in multiple sclerosis and Alzheimer’s disease is described. Finally, the potential role of conditioning as well as the effect of exercise on BBB tight junctions is outlined. There is a body of evidence that regular physical exercise diminishes BBB permeability as it reinforces antioxidative capacity, reduces oxidative stress and has anti-inflammatory effects. It improves endothelial function and might increase the density of brain capillaries. Thus, physical training can be emphasised as a component of prevention programs developed for patients to minimise the risk of the onset of neuroinflammatory diseases as well as an augmentation of existing treatment. Unfortunately, despite a sound theoretical background, it remains unclear as to whether exercise training is effective in modulating BBB permeability in several specific diseases. Further research is needed as the impact of exercise is yet to be fully elucidated.

## Introduction

The blood-brain barrier (BBB) separates the central nervous system (CNS) from the peripheral tissues. In order to maintain homeostasis in the CNS, the BBB controls material, nutrients and cell transfer from the blood to the brain and from the brain to the blood. The BBB restricts the entry of peripheral inflammatory mediators (e.g. cytokines, antibodies, etc.), which can impair neurotransmission [1]. The BBB also participates in the clearance of cellular metabolites and toxins from the brain to the blood [2, 3] and regulates the composition and volume of the cerebrospinal fluid. The BBB is a complex structure consisting of endothelial cells, pericytes, vascular smooth muscle cells, astrocytes, microglia and neurons [2, 4–7] as is located between the brain parenchyma and the vascular system. Interactions between these components have led to the concept of the neurovascular unit, where each cell type contributes to BBB function [8]. The main structures responsible for the barrier properties of the BBB are tight junctions (TJs) [9–15]. The maintenance of adherence, gap and tight junctions between different cell types within the neurovascular unit of the BBB is essential for CNS homeostasis [2, 16]. BBB disturbances commonly occur in neuronal dysfunction, neuroinflammation and neurodegeneration [2].

Numerous pathologic states can cause disturbances in the BBB, including trauma, hypoxia, infection, activation of the clotting system, inflammation, dietary components, environmental toxins and genetic factors [17]. The association between high-grade inflammatory responses such as meningitis, encephalitis, sepsis, local and systemic infections and increased permeability of the BBB for many substances and immune cells has been widely acknowledged [18]. Recently, it became apparent that low-grade systemic inflammation also substantially affects the BBB [19]. Low-grade inflammation affects about 40% of the population in Western countries, as it occurs due to metabolic syndrome, insulin resistance, type 2 diabetes, arterial hypertension, dyslipidaemia and obesity [20–22]. BBB disruption refers to a reduction in barrier tightness and an increase in leakiness. Loss of BBB integrity allows for the entrance of cytokines and immune cells into the CNS, which activates glial cells and causes the alterations in the extracellular environment. Moreover, these changes lead to secondary inflammation and further damage to the BBB, with the leakage of plasma proteins and neurotoxic substances [23, 24].

CNS inflammation determines the severity and disease course of numerous psychiatric and neurological disorders and can be both caused by and result from BBB dysfunction; an inflammatory response in the brain might lead to endothelial cell damage and increased BBB permeability [25]. The permeability of the BBB is altered in many CNS pathologies, including brain injury, ischemic stroke, multiple sclerosis (MS), epilepsy, Parkinson’s disease, Alzheimer’s disease and major depression. A connection has also been reported between BBB impairment and psychiatric disorders such as mood disorders, psychosis, autism spectrum disorder and even chronic sleep disorder [1, 26–29].

It has been well discussed in the literature that inflammatory mechanisms are related to the physiopathology of neuropsychiatric disorders through several mechanisms. Among them are glial activation [30], neuronal damage and degeneration [31], increased oxidative stress [32], reduced neurotrophic support [33], altered neurotransmitter metabolism [34] and BBB disruption [35].

Exercise training is an important behavioural intervention that has numerous beneficial health effects. Epidemiological studies indicate that physical activity leads to systemic adaptations and an elongated health-span, thus time of life in good health, in different human cohorts [36, 37]. Ample evidence suggests that the practice of physical exercise improves some cardiovascular risk factors, such as the percentage of body fat, insulin resistance and high blood pressure, which are associated with increased stiffening of the arteries [38]. Moreover, it has been shown that physical exercise has an impact on inflammation and improved endothelial function by increasing blood flow, which leads to increased shear stress, stimulating the release of nitric oxide.

In this narrative review, we aim to provide a state-of-the-art summary of the influence of exercise on BBB integrity. Consequently, we discuss the influence of physical activity on systemic inflammation, the brain renin-angiotensin and noradrenergic systems and central autonomic function, as well as the kynurenine pathway. There are numerous mechanisms through which long-term physical activity affects the BBB and CNS. It can either diminish inflammation on the peripheral level, thereby reducing the risk of CNS infiltration of immune cells, or protect the BBB through the constitution of its tight junctions.

## Exercise and systemic inflammation

Diabetes mellitus and obesity are exemplar diseases characterised by low-grade inflammation [39, 40]. Notably, inflammation and metabolic dysfunction are frequently associated with oxidative stress in adipose depots [41–43]. Overweight and obese patients with a diagnosis of type 2 diabetes mellitus show increased blood levels of pro-inflammatory cytokines and markers (e.g. IL-1β, IL-6 and TNF-α) [44–46]. These molecules are also increased in the brain, cerebrospinal fluid and in the blood of patients with Alzheimer’s disease or other types of dementia [47]; moreover, an overlap between the pathogenesis of Alzheimer’s disease and diabetes mellitus is evident. Pro-inflammatory cytokines can pass through the BBB and induce stress-activated pathways, promoting brain insulin resistance, mitochondrial dysfunction [19] and the accumulation of neurotoxic beta-amyloid (Aβ) oligomers [48] leading to synaptic loss, neuronal dysfunction and cell death. Increased ceramide production caused by dysregulated lipid metabolism also occurs with insulin resistance [49]; these molecules can pass through the BBB, induce pro-inflammatory reactions and disturb brain insulin signalling [50].

Physical training may be crucial in preventing as well as diminishing this damage. Regular physical exercise reinforces antioxidative capacity, reduces oxidative stress and has anti-inflammatory effects. It improves endothelial function and might increase the density of brain capillaries. Physical training can further counteract dyslipidaemia and reduce increased ceramide levels [51, 52], and it may have a suppressive effect on the BBB damage cycle. Abd El-Kader et al. [53] presented data that both endurance and strength training can potentially alleviate the inflammatory state due to a reduction in TNF-α levels in type 2 diabetes mellitus patients. De Senna et al. [54] have also demonstrated that exercise improves the structural components of the BBB in diabetic rats. Regular exercise training induces a reduction in adipose tissue-derived pro-inflammatory cytokines like IL-6, TNF-α and MCP-1[55–58], which are associated with low-grade systemic inflammation and provides reduction in whole-body insulin resistance [59].

Importantly, physical activity is able to decrease inflammation independently from weight loss, through a reduction in inflammatory cytokines release from skeletal muscles. Similar to adipose tissue as well as immune cells, skeletal muscle, which is the largest organ in the human body, produces and releases inflammatory cytokines such as IL-4, IL-6, IL-8, IL-15 and TNF-α [60–62]. These cytokines are therefore also called myokines [60, 63]. Handschin and Spiegelman [64] hypothesised that physical activity can broadly suppress myokine expression through the upregulation of skeletal muscle peroxisome proliferator activated receptor 1α. The upregulation of this receptor is induced by physical activity [65]. In a study by Aronson et al. [66], C-reactive protein (CRP) concentrations decreased continuously with increasing levels of physical fitness. Increases in circulating IL-6 have been observed after performing exercise without muscle damage; this in turn reduces pro-inflammatory cytokines via the stimulation of the anti-inflammatory cytokines IL-1ra and IL-10 [61].

A recent study by Chupel et al. [67] showed that physical exercise can maintain BBB integrity. The anti-inflammatory effect of combined exercise training and taurine supplementation on peripheral markers of BBB integrity, inflammation and cognition in 48 elderly women was investigated. The results showed a reduction in TNF-α and IL-6, as well as a reduction in the IL-1β/IL-1ra, IL-6/IL-10 and TNF-α/IL-10 ratios in the combined exercise training group. Interestingly, the improvement in cognitive function was reported only in women subjected to both exercise and taurine augmentation.

## Exercise and kynurenine pathway

Brain and peripheral inflammatory states can also promote BBB failure through tryptophan (TRP) catabolism as a consequence of kynurenine pathway activation, which is also connected with glutamatergic excitotoxicity. Tryptophan is metabolised via several pathways, the main one being through kynurenine (KYN) [68], which is involved in several conditions including cancer, inflammatory disorders, diabetes mellitus, neurological and neurodegenerative diseases. Tryptophan is degraded into kynurenine by the enzyme indoleamine 2,3-dioxygenase (IDO) [69, 70]. Kynurenine is then metabolised into neuroprotective kynurenic acid (KYNA) by kynurenine aminotransferases (KATs) or into neurotoxic products such as quinolinic acid (QUIN) or anthranilic acid. QUIN is a selective agonist of *N*-methyl-d-aspartate (NMDA) receptors, and a potent neurotoxin due to its ability to induce the production of reactive oxygen species. KYN reduces the activity of natural killer cells, dendritic cells or proliferating T cells, whereas KYNA promotes monocyte extravasation and controls cytokine release. High levels of QUIN have been associated with neuronal excitotoxicity, whereas KYNA has been reported as a neuroprotective factor [71] through its inhibitory action at glutamatergic excitatory synapses [72]. Among the kynurenine pathway metabolites, QUIN is likely to be one of the most important in terms of biological activity and toxicity [73]. Both KYNA and QUIN have been found to be dysregulated in major depressive disorders and schizophrenia [74]. Pro-inflammatory mediators such as TNF-α can promote QUIN production [75], while IL-1β potentiates quinolinate-mediated excitotoxicity [68]. This is particularly important in situations involving macrophage infiltration across the BBB. In CNS inflammatory states, IDO is mainly activated in microglial cells, which preferentially metabolise tryptophan into the NMDA receptor agonist QUIN. The most potent activator of IDO is interferon gamma (IFN-γ) [70, 76]. Therefore an imbalance in type 1/type 2 immune responses, associated with an astrocyte/microglia imbalance, leads to serotonergic deficiency and glutamatergic overproduction. Astrocytes are further strongly involved in the re-uptake and metabolic conversion of glutamate. A reduced number of astrocytes could contribute to both diminished counter-regulation of IDO activity in microglia and altered glutamatergic neurotransmission. Binding of excess glutamate to dysregulated BBB endothelial cell ionic NMDA receptors and metabotropic glutamate receptors can increase intracellular Ca^2+^-dependent oxidative stress and BBB permeability by increasing Ca^2+^ influx and release from endoplasmic reticulum stores, respectively.

Skeletal muscle has recently been added [77] to the list of tissues that contribute to kynurenine pathway metabolism. This happens in the setting of exercise training, which enhances KAT gene expression and the conversion of toxic KYN to neuroprotective KYNA. The neuroprotective effect of KYNA is generally attributed to its antagonistic action on NMDA receptors.

It has been found by András and colleagues [78] that a high glutamate level diminishes the function of the BBB via endothelium-expressed NMDA receptor-dependent occludin phosphorylation. KYNA is not only an endogenous NMDA receptor blocker but also a non-competitive inhibitor of the α7-nicotinic acetylcholine receptor [68, 79, 80]; through this mechanism, KYNA can decrease glutamate release [81]. In this manner, KYNA can reduce pathological glutamate levels and protect the structure of the BBB [78]. The effect of exercise training on the BBB highlights an important mechanism of inter-organ cross-talk as kynurenine accumulation can be suppressed by activating its clearance in exercised skeletal muscles.

## Exercise and the renin-angiotensin-aldosterone pathway

There is a linkage between angiotensin II (Ang II), one of the renin-angiotensin-aldosterone factors, and the disruption of the BBB, especially in hypertension states [82–84]. Although Ang II is known as a cardiovascular mediator, with a primary role in the regulation of blood pressure and fluid homeostasis [85], it performs as an immune system modulator as well. Ang II can initiate inflammation by indirect promotion of vascular permeability and the recruitment of inflammatory cells [86]. Ang II may also activate both innate and adaptive immunity [86, 87].

Ang II directly modulates transcytotic and paracellular permeability in BBB endothelial cells and could contribute to the pathophysiology of hypertensive encephalopathy. Both circulating Ang II via Ang II type 1 receptors (AT_1_) on the endothelium and locally synthesised Ang II can result in vascular dysfunction and microglial activation, increase the production of reactive oxygen species, disrupt endothelial nitric oxide synthase activity and intensify pro-inflammatory cytokine synthesis, which are important factors for sympathoexcitation centres such as the paraventricular nucleus (PVN) of the hypothalamus and the rostral ventrolateral medulla (RVLM) in neurogenic hypertension [88–90]. However, one study has indicated that inhibition of AT_1_ blocks hypertension-related increases in cell permeability and cerebral oedema, even in the absence of lowered blood pressure [91]. This suggests a role for the renin-angiotensin-aldosterone system and Ang II in modulating BBB function. BBB disruption not only facilitates Ang II access but also allows circulating inflammatory cells to enter into the brain parenchyma, contributing to further microglial activation and inflammation in autonomic areas such as the PVN of the hypothalamus and the RVLM [89, 90]. These responses alter neurovascular coupling, dysregulate cerebral perfusion and markedly augment neuronal discharge, thus exacerbating sympathoexcitation in hypertensive animals [83, 92, 93].

Physical exercise has been shown to be highly efficient in reducing the harmful effect of hypertension on BBB leakage in autonomic brain areas, which strongly correlates with the improvement of both parasympathetic and sympathetic control of cardiovascular parameters, even in the persistence of hypertension. Recent studies have established the efficacy of aerobic training to downregulate the brain renin-angiotensin system and correct autonomic dysfunction in spontaneously hypertensive rats [94, 95]. These rats, after 2 weeks of physical exercise, have been reported to maintain normal angiotensinogen expression within autonomic areas, which is correlated with reduced sympathetic outflow to the heart and vessels and precedes a partial fall in arterial pressure [96]. Exercise training restores the balance between the excitatory and inhibitory neurotransmitters as well as between pro- and anti-inflammatory cytokines, and attenuates oxidative stress in the PVN [97]. Trained spontaneously hypertensive rats demonstrate an instant normalisation of the baroreceptor reflex control of heart rate that coincides with a marked reduction in oxidative stress and inflammation in the hypothalamic PVN [98]. Similar training-induced effects have also been observed in other autonomic areas [94, 95]. It is already known that acute physical activity induces sympathetic modulation [99]. Conversely, regular exercise may be associated with progressive sympathetic withdrawal and increasing parasympathetic dominance as a result of adaptations of the peripheral and central regulatory systems [99]. It has been shown that hypertension is characterised by autonomic impairment, BBB leakage and Ang II-induced neuronal activation and that exercise training is highly effective at preventing Ang II-induced effects and improving autonomic function [100]. Physical training changes the tissue Ang II content as well and suppresses microglial activation, crucial factors for both the maintenance of BBB integrity and the normalisation of autonomic control of the circulation in hypertensive individuals. According to some authors, there may be a system of global control over the permeability of the cerebral microvasculature via the cholinergic nervous system [101]. Due to the fact that exercise induces a shift toward parasympathetic dominance, this mechanism may also be favoured.

There is an interaction between neurotransmitters, pro-inflammatory cytokines and enhanced oxidative stress in the PVN which play a key role in sympathetic regulation of blood pressure [95]. In spontaneously hypertensive rats, reactive oxygen species in the RVLM are known to enhance glutamatergic excitatory inputs and impair GABAergic inhibitory inputs to the RVLM, resulting in increased sympathoexcitatory input to the RVLM from the PVN [102]. Among the known pressor agents, Ang II and glutamate play pivotal roles in the brain centres involved in blood pressure control in both normotensive and spontaneously hypertensive rats [103]. The link between brain angiotensinergic and glutamatergic signalling has been demonstrated by Vieira et al. [104]. The major sympathetic output pathway for the tonic and reflex control of blood pressure, which uses glutamate as a transmitter, arises in the RVLM [105]. Injection of Ang II into the RVLM of unanaesthetised rats exaggerates the pressor response to glutamate. Additionally, it has been speculated that Ang II takes part in glutamate pressor responses via a presynaptic increase in glutamatergic input into the RVLM [106]. Referring to this, KYNA (a glutamate antagonist) is thought to be a hypotensive agent. Mills et al. [107] have reported that intrathecal KYNA administration decreases blood pressure, especially in anesthetised spontaneously hypertensive rats and stroke-prone spontaneously hypertensive rats, with a less noticeable effect in normotensive rats.

It can be assumed that in hypertension states, there is an ability of physical exercise to correct sympathetic hyperactivity, reduce Ang II availability, decrease oxidative stress and inflammation in the PVN and RVLM and restore BBB integrity.

## Exercise and brain noradrenergic system

During physical exercise, it is well known that peripherally circulating epinephrine and norepinephrine (NE) activate β-adrenoceptors of the vagus nerve afferents projecting to the locus coeruleus, which plays a pivotal role in the regulation of autonomic activity and cognitive functions [108–112]. The optimal stimulation of locus coeruleus might be a crucial element of modulating cognitive function with exercise [113]. Brain NE is also reported to suppress the inflammatory gene transcription. It is clear from the findings of Hetier et al. [114] and Frohman et al. [115] that anti-inflammatory action of NE is exerted via microglia and astrocytes β2-receptors. Immune cells express both types of receptors, and T and B lymphocytes express β2-receptors almost exclusively [116]. Engagement of β2-receptors activates a cascade of signaling intermediates, including cyclic adenosine monophosphate **(**cAMP) and protein kinase A, which leads to the phosphorylation of cellular proteins [116]. NE also promotes a shift of Th1/Th2 balance toward Th2 response by activating β2-receptor [117]. Other anti-inflammatory effects of NE have also been reported, including the suppression of inducible nitric oxide synthase, interleukin-1b, tumor necrosis factor α and intercellular adhesion molecule-1. Attention is also devoted to the fact that NE enhances brain-derived neurotrophic factor production, which plays an important role in neuronal survival, neuroplasticity and neurogenesis [37, 118]. This interaction of brain-derived neurotrophic factor is mediated by β1/β2, and α2-adrenergic receptors and shares similar cellular pathways with anti-inflammatory NE action [118–120].

Neurons metabolize glucose primarily in the pentose phosphate pathway in order to produce adenosine triphosphate (ATP) to support brain functions, including glutathione regeneration, which protects the neuron from reactive oxygen species (Bélanger et al. 2011), and consequently stands for proper BBB functioning. Apart from using glucose as the first choice, lactate is also used by the brain as a fuel for neurons [121, 122]. This process occurs in the astrocytes, where the recruitment of energy from their glycogen stores [123] is facilitated by noradrenergic stimulation of the astrocytes’ β-adrenoceptors signaling them to convert glycogen to lactate [124, 125], which is then transported to the neurons [126]. In response to physical exercise, the lactate production by skeletal muscles increases [127, 128], as does the expression of the lactate transporter monocarboxylate transporter 1 at the BBB [129–131]. Lactate produced during exercise is also reported to increase levels of growth factors important to angiogenesis, neurogenesis, calcium signaling, axonal myelination, synaptic plasticity and memory formation [132–134]. Energetic insufficiency in neurons due to inadequate lactate supply is implicated in several neuropathologies, including attention-deficit/hyperactivity disorder [135–137].

## Exercise in multiple sclerosis

MS has recently attracted the interest of numerous researchers [138] regarding the application of physical activity. In MS, there is an extrinsic BBB disruption pattern with the initial injury in the blood vessels, allowing T and B cells to cross the BBB [139] with the exudation of fibrin, which causes an inflammatory reaction leading to demyelination [140], immune cell infiltration and axonal damage. In accordance with a study by Mokhtarzade et al. [141], it has been shown that 8 weeks of exercise training normalises the concentration of particular BBB permeability markers in MS patients, including S100 calcium-binding protein B (S100B). Additionally, according to White et al. [142], exercise is potentially able to counteract the imbalance between the pro-inflammatory Th1 cytokines and the anti-inflammatory cytokines (for example IL-10) by enhancing anti-inflammatory mechanisms in MS patients.

According to Rossi et al. [143], exercise can protect from inflammation-induced neurodegenerative synaptic and dendritic alterations in the experimental autoimmune encephalomyelitis mouse model of MS. In this study, exercise has been shown to increase synaptic density and growth in the hippocampus [143]. In animal model of MS, positive effects of exercise upon cognition are also reported [144, 145]. Moreover, an increase in neurotrophins [145] and brain-derived neurotrophic factor [146] in response to exercise has been found.

However, there is still an urge for human researches. The analyses of studies indicate that ideally both aerobic and resistance exercises may benefit persons with MS. A substantial increase in hippocampal volume has been reported in MS subjects randomized to an aerobic exercise program compared with a nonaerobic-trained control [147]. Physical fitness exercise correlates with improved cognitive function in persons with MS [148, 149]. Another finding indicates that cardiorespiratory fitness in patients with MS predicts neuronal plasticity [150] and increased gray matter volume, better white matter integrity and improved performance on test of information processing speed [151]. There is some evidence for neuroprotective effects of exercise in humans with MS, comparing aerobic to aerobic plus resistance exercise training resulting in decreases in anti-inflammatory cytokines [148].

Based on these findings, it can be postulated that physical exercise reduces inflammation and, as a consequence, BBB impairment, and thus has a protective effect on CNS in MS patients. If physical activity is able to enhance BBB stability in these particular patients, we can predict that it will improve BBB function in general. Nevertheless, studies with larger group of patients and clearly developed exercise protocols are needed to include physical activity sessions as a standard of care therapeutic procedures.

## Exercise in Alzheimer’s disease

In Alzheimer’s disease, the integrity of BBB may be disordered according to accumulation of reactive oxygen species activating metalloproteinases which leads to breakdown of BBB through destruction of basement membrane and tight junctions [152], as well as due to the accumulation of cholesterol metabolism [152], and impaired insulin signaling [153]. In the prevalence of BBB maintenance complications, the amplified Aβ microvessels deposits process begins. As Aβ fibrils accumulate, it results in a cascade of significant brain degeneration and atrophy of hippocampal and cortical structures. BBB dysfunction during Alzheimer’s disease influences Aβ clearance and endothelial transport, impairs endothelial cell and pericyte functions, affects TJ integrity, activates glial cells and facilitates the recruitment of leukocytes in the brain [154–158].

Physical activity can improve Aβ clearance by upregulating Aβ transporters and reduce the accumulation of Aβ peptides in the brain parenchyma by accelerating interstitial fluid drainage, which diminishes neuroinflammation [159]. Although a direct effect on specific Alzheimer’s disease pathology is still unproven [160], one research documented a reduction in tau in cerebrospinal fluid as a result of exercise in older adults with mild cognitive impairment [161]. Exercise, primarily aerobic, improves cognitive function in several patient populations, including Alzheimer’s disease, elderly people at risk for cognitive impairment and healthy older adults [162, 163]. In addition, both animal and humans studies suggest that physical activity may have a role in modifying the disease process and maintaining cognitive function in Alzheimer’s disease [11]. Physical activity interventions has a positive overall effect on cognitive function, and the effect is driven by interventions that included aerobic exercises independent of the type of dementia [164], which has been recently proved in meta-analysis conducted by Öhman et al. [165].

Further studies shall unveil if/how neuroinflammation and BBB dysfunction are related to cognitive function impairment and overall Alzheimer’s disease progression. Then, specific physical activity protocols may be developed to support patients care.

## Exercise-induced conditioning

Physical activity acutely elevates the release of adrenaline, cortisol, growth hormone, prolactin and other factors with immunomodulatory effects [166]. Consequently, very high intensity exercise can trigger systemic inflammation, a subsequent immunodepression and thus a higher risk of infections [167]. According to Roh et al. [168], moderate- and/or high-intensity exercise may induce higher oxidative-nitrosative stress than low-intensity exercise.

While acute intense exercise provokes a spike in the activity of inflammatory cells (like leukocytes) and plasma CRP concentrations, repeated bouts of submaximal intensity exercise induce adaptive mechanisms that can counteract inflammation in the long term [169]. These changes are measurable as reduction in the concentration of inflammatory mediators such as CRP, IL-6 and TNF-α, while enhancing the productions of the anti-inflammatory IL-10 [170].

There are some contrasting studies regarding the influence of physical exercise on BBB integrity. S100B is considered to be the best indicator of BBB permeability [168, 171, 172] and can even predict the severity of brain injury [168, 173]. Elevated S100B levels have been recorded following exercise and are mostly attributed to either an elevation in BBB permeability or head trauma [174–176]. Increased serum concentrations of S100B have therefore been used to associate CNS pathology with BBB dysfunction. However, even in the absence of head trauma, it appears that the BBB may be compromised following exercise, with the severity dependent on exercise intensity. According to Sharma et al. [177], short-term forced swimming exercise increases the permeability of the BBB in specific brain regions in rats, likely mediated through serotonin via 5-HT2 receptors. Intense exercise has the potential to increase S100B and induce BBB functional deterioration without causing structural brain damage subsequent to a free radical-mediated impairment in dynamic cerebral autoregulation [178].

After acute exercise, high levels of myokines are secreted by the skeletal muscle, exerting a variety of endocrine effects. The induction of myokines like myostatin, IL-7, decorin and leukaemia inhibitory factor is involved in the regulation of muscle hypertrophy and may play a role in the restructuring of skeletal muscle as a response to exercise [179].

Exercise reduces the expression of Toll-like receptors at the surface of monocytes; these receptors have been implicated as mediators of systemic inflammation [180]. Muscle function, inflammation and exercise are hence intrinsically linked in a complex manner [64, 181]. The induction of the beneficial versus detrimental effects of physical activity therefore seems to be highly context-specific. For example, IL-6 was originally classified as a prototypical pro-inflammatory cytokine, although anti-inflammatory properties have also been described [182]. Some studies have provided data showing an increase in IL-6 concentrations directly after physical activity, suggesting that IL-6 was released from the muscle mass and acts as a myokine, rather than a typical pro-inflammatory cytokine [183]. Besides the production of IL-6 in activated immune cells, the systemic elevation of IL-6 in patients with metabolic diseases has strengthened the link between IL-6 and inflammation. In stark contrast, however, exercise-induced elevations in IL-6 plasma levels lead to increased circulating levels of several potent anti-inflammatory cytokines such as IL-1ra and IL-10, and also inhibit TNF-α production, suggesting that IL-6 may also have anti-inflammatory properties [184, 185]. Skeletal muscle fibres also express and release IL-6 during and after exercise [186–189]. IL-6 production is likewise boosted in connective tissue, the brain and adipose tissue post-exercise [60].

Thus, in the case of exercise, IL-6 exerts anti-inflammatory effects [63]. This mechanism may explain why lower baseline levels of pro-inflammatory cytokines are present in those people who are most physically active [190]. Cells respond to the stressful stimulus of exercise by activating pathways to abolish it; in this case, by increasing the expression of enzymes such as superoxide dismutase and glutathione peroxidase or by increasing levels of anti-oxidative peroxiredoxins. A likely mediator of this response are proteins related to the NFκB pathway, which can activate the gene expression of anti-oxidative proteins and show enhanced DNA binding around 2 h after acute exercise [191, 192]. Consequently, physical activity reduces the production of reactive oxygen species and enhances the anti-oxidative defence [193, 194]. In skeletal muscle in particular, repeated moderate intensity exercise improves the anti-oxidative capacity by upregulating endogenous anti-oxidative molecules [195, 196].

Thus, we can assume that brief, intense physical activity increases the permeability of the BBB [177] and induces inflammation, but long-term regular exercise may have a protective role on BBB integrity and activate anti-inflammatory pathways.

## Physical Exercise and BBB tight junctions

The BBB is composed of endothelial cells forming a selective vascular network through the expression of tight junction (TJs) complexes, which are defined as molecules that interact in the extracellular junctional space or as molecules that act as anchors within the endothelial cell to create the BBB [174]. Tight junctions between brain endothelial cells are constituted by three major transmembrane proteins: occludin, claudins and junction associated molecules, as well as several cytoplasmic proteins including zonula occludens [197]. Brain endothelial TJs express claudin-3 and -5 and possibly claudin-12 [198, 199]. Claudin-5 has been shown to actively contribute to BBB integrity [200]. Many studies have indicated that occludin, claudin-3 and claudin-5 are involved in BBB genesis [199, 201] and the control of paracellular permeability [202–205].

TJs can be regulated by the activation of various receptors of vasoactive compounds (bradykinin and Ang II) as well as adhesion molecules or reactive oxygen species. Intercellular adhesion molecule 1 (ICAM-1), vascular cell adhesion molecule 1 (VCAM-1) and platelet and endothelial cell adhesion molecule 1 (PECAM-1), members of the immunoglobulin superfamily, actively contribute to the firm adhesion and/or migration of leukocytes into the CNS through the cytokine-activated brain endothelium [206, 207]. Agents released from most of the cells of the neurovascular unit during pathology can modulate brain endothelial tight junctions, with several inflammatory mediators exacerbating BBB permeability; only a few agents are able to counter or reverse this course [1]. The prolonged presence of oxidative/inflammatory factors can result in changes to or the loss of tight junctions and integrins (e.g. β1, αv and α6 integrins), leading to senescence and detachment-mediated cell death [23, 208]. It is known that both IFN-γ and TNF-α can alter BBB permeability by affecting the cellular distribution of junctional adhesion molecules and by crucial upregulating the expression of ICAM-1 and VCAM-1 [209–211].

Changes in BBB permeability are connected with alterations in occludin expression [212] and endothelial barrier function [202]. The selectivity of claudin-5 expression in brain endothelial cells suggests that it is essential for BBB function [213], as it has been demonstrated that claudin-5 participates in modulating permeability to ions as well as macromolecules [214]. Several studies have indicated that altered permeability of the BBB is accompanied by decreased claudin-5 expression [213].

Attention should be also devoted to recent research by Souza et al. [215] regarding the impact of physical exercise, which re-establishes the expression of TJ proteins such as occludin and claudin-4 in the CNS to basal levels and inhibits the expression of PECAM-1. This study was based on experimental autoimmune encephalomyelitis, a mouse model of MS. The study suggests that physical exercise maintains the integrity of the BBB by preserving tight junctions. Additionally, Schreibelt et al. [216] demonstrated that physical exercise in MS preserves the levels of claudin-4 and occludin in the spinal cord of mice, by inhibiting the production of reactive oxygen species and the induction of oxidative stress. Moreover, some studies have provided data that inhibiting glycogen synthase kinase-3β promotes TJ stability in brain endothelial cells by extending the half-life of occludin and claudin-5 and increasing their levels, which does not involve their gene regulation [217]. A study by Isla et al. [218] showed a favourable effect of voluntary exercise involving a reduction in glycogen synthase kinase-3β recruitment, resulting in protection of the BBB through TJs.

The inhibition of glycogen synthase kinase-3β has also anti-inflammatory effect on brain endothelial cells [219]. In addition to the barrier-enhancing role of glycogen synthase kinase-3β inhibitors, it gives a promise to their utility in repair and protection of the BBB.

## Clinical significance of exercise

Human organism’s response to repeated high levels of physical exercise provides an integration of cells, organs and organ systems in order to minimize homeostatic disruptions during and after exercise, which is a stress stimuli. Regular moderate–high levels of exercise may be perceived as physiological as due to the sedentary lifestyle, the reduction of the maximal capacity of organ systems is observed, leading to several pathological processes. Therefore, individuals with high levels of exercise capacity present decreased prevalence of pathological chronic diseases and of mortality [36].

**Fig. 1:**
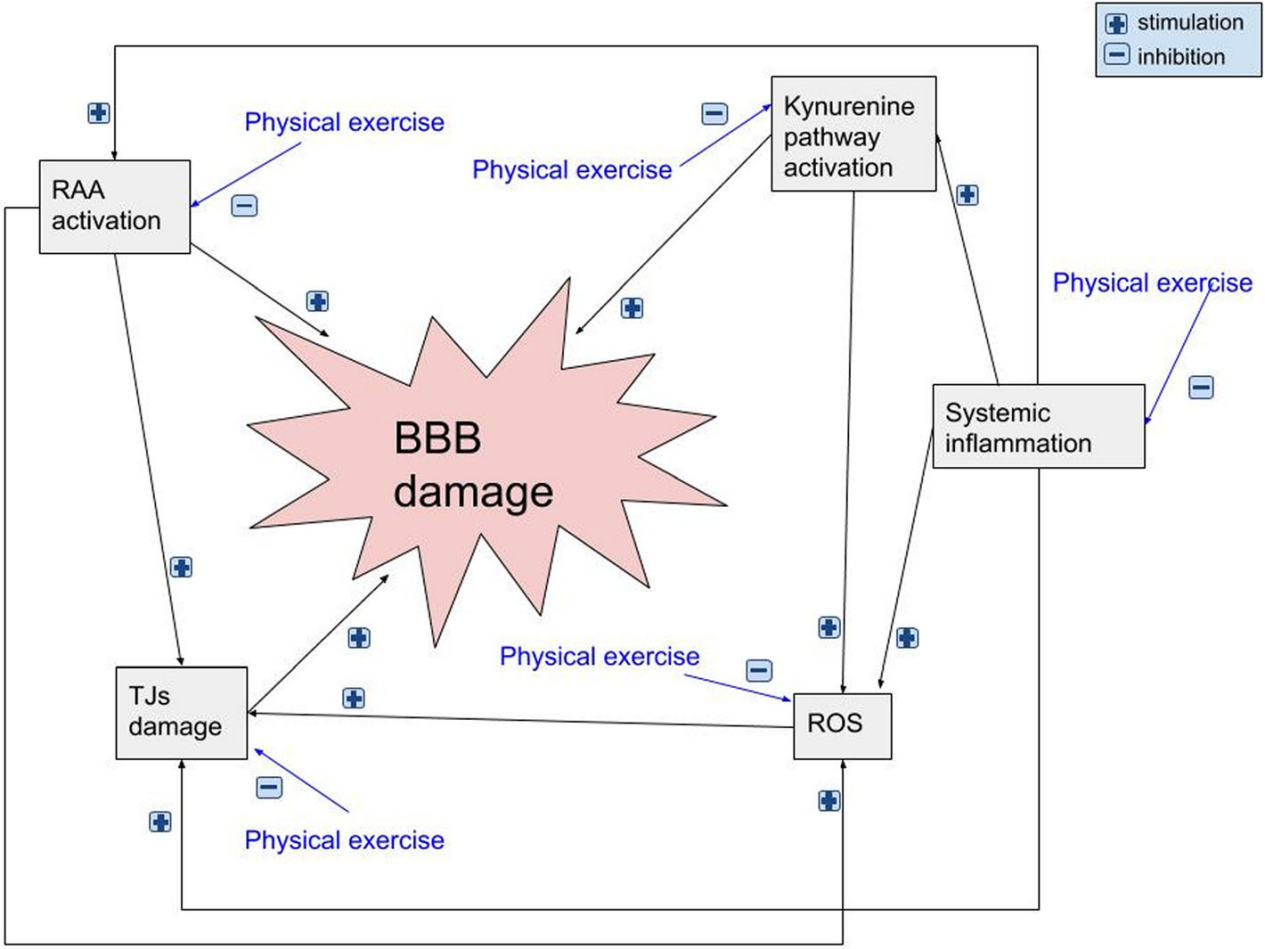
A low-grade systemic inflammation observed in metabolic syndrome, insulin resistance, type 2 diabetes, arterial hypertension, dyslipidaemia and obesity contributes to BBB damage via pro-inflammatory cytokines inducing kynurenine pathway leading to neurotoxic process on BBB and its permeability. In inflammatory states, ROS are also produced and may impair TJs structure and function causing the direct damage of BBB. RAA system activation due to the systemic inflammation increases the destruction of TJs and production of ROS leading to further damage of TJs and BBB. Physical exercise counteracts obesity and diminishes the low-grade systemic inflammation, production of ROS and changes kynurenine pathway metabolism course into neuroprotective agents as well as downregulates brain RAA system, which all leads to BBB protection. *BBB* blood-brain barrier, *ROS* reactive oxygen species, *TJs* tight junctions, *RAA* renin-angiotensin-aldosterone

Physical inactivity is a primary cause of several chronic diseases [220]. Physical exercise improves cerebrovascular, metabolic and endothelial function, which reduces oxidative stress and neuroinflammation, contributing to improved neuronal function [221–223]. Moreover, exercise is associated with increases in levels of brain-derived neurothropic factor and insulin-like growth factor 1 [160, 223, 224]. The accumulating evidence reinforces the position that regular aerobic [225, 226] and, with less evidence of effectiveness, resistance training [227] offer a powerful tool to cope with biologic aging of selected central nervous system functions. Figure 1 depicts the convergence of multiple pathways, activated by exercise, on a final, anti-inflammatory common pathway.

In the general population, exercise improves attention, processing speed, memory and executive functioning. Exercise also increases hippocampal volume and white matter integrity in healthy older adults [228, 229]. It is a behavioral intervention that shows great promise in alleviating symptoms of some mental disorders such as depression [230] and can significantly improve positive symptoms, negative symptoms and social functioning in patients with schizophrenia [231–233].

Apart from playing a role in diminishing the diseases associated with leaky BBB, physical exercise is known to induce beneficial effects in different systems, e.g. the cardiovascular, muscular, metabolic, neural, respiratory and thermoregulatory [234–238]. Physical training results in an increase in the concentration of the anti-inflammatory cytokine IL-10 and a decrease in the pro-inflammatory cytokines IL-1β and TNF-α [239]. Exercise training has also been reported to ameliorate the inflammatory profile in patients after a myocardial infarction by enhancing the expression of the anti-inflammatory cytokine IL-10 [240]. According to Lin et al. [241], IL-10 improves properties of the BBB in a rat model of severe acute pancreatitis by attenuating the downregulation of claudin-5 expression and the impairment of tight junctions and by anti-apoptotic effects on brain microvascular endothelial cells. Harris et al. [242] have shown that exercise modulates immunological and exerts anti-inflammatory effects in the CNS, such that depression-like symptoms are reduced. Moreover, exercise reduces the expression of Toll-like receptors on the surface of monocytes [180, 243–245], which may represent a beneficial effect as Toll-like receptors are responsible for mediating the capacity of monocytes and macrophages to produce inflammation [246–248].

The accumulating evidence reinforces the position that regular aerobic, and possibly also resistance training, plays an important role in maintenance of healthy structures and functions of the human body [37]. Being a valuable component in the clinical management of a variety of diseases, it is recommended for these purposes in numerous evidence-based clinical guidelines [249, 250]. There is a current need of novel nonpharmacological strategies such as physical exercise that can provide valuable adjunctive treatment but further studies are warranted to decipher the exact role physical exercise play in some neuroinflammatory diseases.

## Conclusions and future directions

**Fig. 2:**
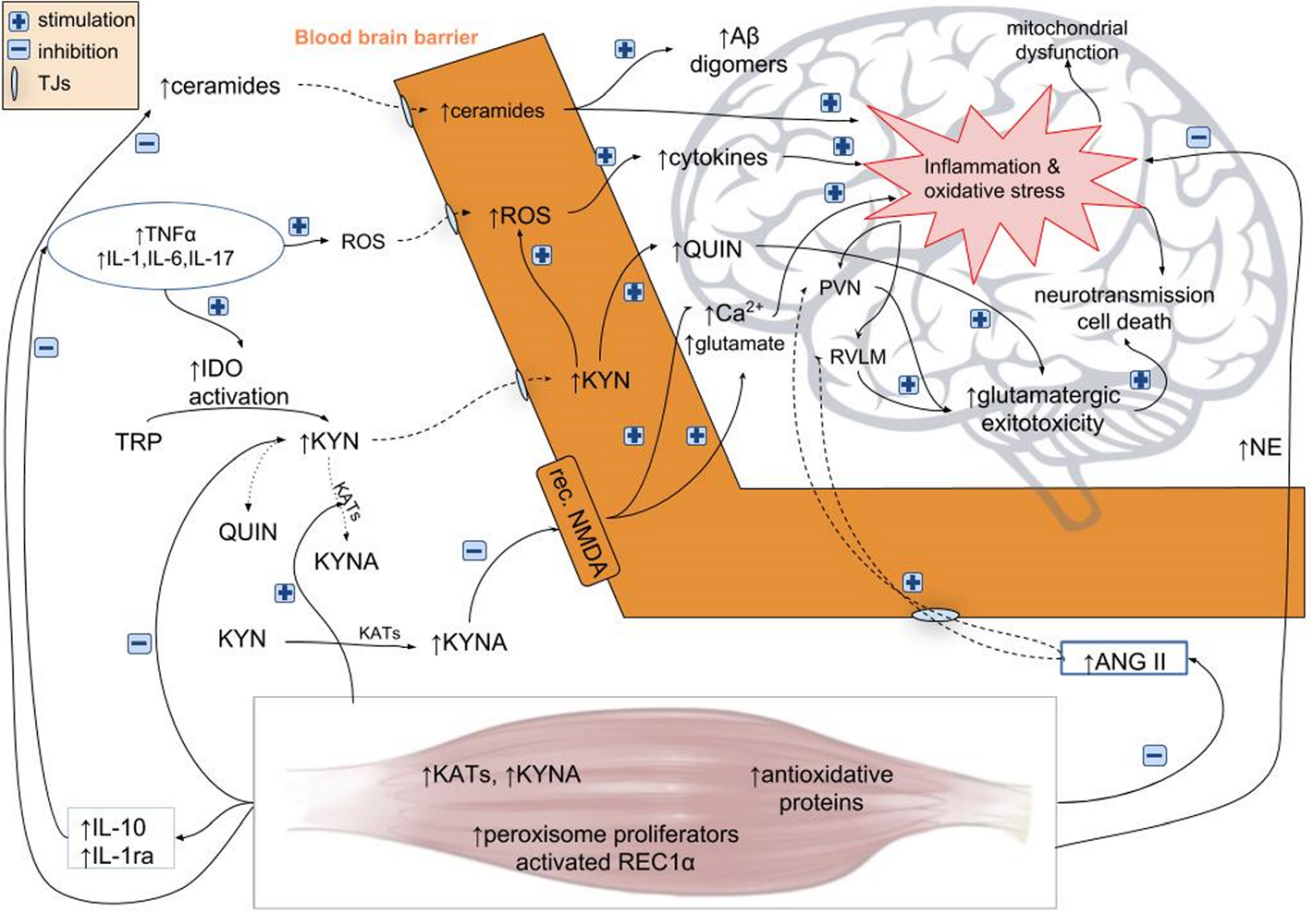
In systemic low-grade inflammatory states, cytokines can stimulate ROS production destroying tight junctions and increasing BBB permeability. Cytokines can also activate IDO catalyzing degradation of tryptophan into KYN. KYN can be transformed into neuroprotective KYNA by KATs enzyme or into neurotoxic products, mainly QUIN, which stimulates NMDA receptors and leads to glutamatergic overproduction increasing Ca^2+^ influx and BBB breakage. Low-grade inflammation in insulin resistance causes lipid dysregulation and increased ceramide production and its pass through the BBB, intensifying brain inflammation and promoting Aβ production. In leaky states of BBB, TJs lose their function and pro-inflammatory factors can easily pass through BBB leading to its further damage. The presence of inflammation and increased oxidative stress in brain impair significantly mitochondrial and neuronal functions causing cell death. During BBB disruption, facilitated Ang II access can initiate inflammation by promotion of vascular permeability via AT_1_ receptors, rising the recruitment of inflammatory cells, ROS production, microglial activation and inflammation in autonomic areas such as the PVN and the RVLM, which potentiate glutamatergic toxicity. Physical activity enhances KAT gene expression and the conversion of toxic KYN to neuroprotective KYNA, which protects BBB. During physical activity, the muscles release of anti-inflammatory cytokines IL-1ra and IL-10, which can itself reduce the concentration of pro-inflammatory cytokines (TNF-α, IL-1, IL-6, IL-17) as well as by upregulation of skeletal muscle peroxisome proliferator activated receptor 1α. NE released during physical activity acts via microglia and astrocytes β2-receptors and lymphocytes β2-receptors reducing the neuroinflammation. Physical training also diminishes the tissue Ang II content and can suppress microglial activation in the PVN and the RVLM. Physical exercise reinforces antioxidative capacity by upregulating endogenous anti-oxidative molecules, reduces oxidative stress and ceramide levels with a suppressive effect on the TJs and BBB damage cycle. *Ang II* angiotensin II, *AT*_*1*_ angiotensin II type 1, *BBB* blood-brain barrier, *IDO* indoleamine 2,3-dioxygenase, *KYN* kynurenine, *KYNA* kynurenic acid, *KATs* kynurenine aminotransferases, *NE* norepinephrine, *NMDA N*-methyl-d-aspartate, *PVN* paraventricular nucleus, *QUIN* quinolinic acid, *RVLM* rostral ventrolateral medulla, *ROS* reactive oxygen species, *TJs* tight junctions, *TRP* tryptophan

In this review, a theoretical framework on the crosstalk between physical exercise and BBB permeability is presented. In our model, physical exercise influences the BBB through a number of anti-inflammatory effects and leads to a reduction in lesions and vascular permeability (Fig. 1). BBB breakdown generally culminates in neuronal dysfunction, neuroinflammation and neurodegeneration. The pathogenesis of numerous diseases has been recently shown to be inflammatory in nature, and there is increasing interest in non-pharmacological, alternative methods of treatment. Regular physical exercise diminishes BBB permeability as it reinforces anti-oxidative capacity, reduces oxidative stress and has anti-inflammatory effects. It improves endothelial function and might increase the density of brain capillaries (Fig. 2).

Although physical exercise has positive effects on brain function, there have been only a few studies assessing the long-term consequences of physical activity on BBB permeability. To our knowledge, there are only two studies that have directly explored the nexus between regular exercise and BBB parameters. Mokhtarzade and colleagues [141] indicated an improvement in BBB leakage markers, including S100B, after 8 weeks of exercise training in MS patients. Souza and colleagues [215] demonstrated in the experimental autoimmune encephalomyelitis mouse model that a 4-week-long exercise program resulted in immunomodulatory and antioxidant effects as well as in the maintenance of BBB integrity by preserving its tight junctions. Consequently, despite sound theoretical background, it remains so far unclear as to whether exercise training is effective in modulating BBB permeability in specific diseases. In particular, the potential magnitude of BBB function improvement or restrain of disease-related BBB deterioration has to be investigated. Moreover, the influence of different kinds exercise and the quantity of physical activity required to induce meaningful responses remain to be clarified.

Novel imaging modalities are available to study disruption of the BBB in humans starting from large permeability leaks observed in MS to more subtle changes in chronic vascular disease and dementia [251]. Consequently, future studies should link BBB permeability with clinical presentation of the patients. Moreover, further research is required to determine the significance of BBB protection with physical exercise in relation to functional benefits for patients such as improved cognition and daily functioning. Finally, the effects of exercise on interactions between neuroinflammatory status and BBB permeability shall be defined in experimental studies.

Therapeutic interventions targeting disturbances in BBB function might therefore have a promising effect in a broad spectrum of disorders. Physical training can be emphasised as a component of prevention programs developed for patients to minimise the risk of the onset of inflammatory diseases as well to complement treatment. More research is needed as the impact of exercise on BBB function is yet to be fully elucidated.

## Abbreviations

Ang II: Angiotensin II
AT_1_: Angiotensin II type 1
ATP: Adenosine triphosphate
Aβ: Beta-amyloid
BBB: Blood-brain barrier
cAMP: Cyclic adenosine monophosphate
CNS: Central nervous system
CRP: C-reactive protein
ICAM-1: Intercellular adhesion molecule 1
IDO: Indoleamine 2,3-dioxygenase
IFN-γ: Interferon gamma
KATs: Kynurenine aminotransferases
KYN: Kynurenine
KYNA: Kynurenic acid
MS: Multiple sclerosis
NE: Norepinephrine
NMDA: *N*-methyl-d-aspartate
PECAM-1: Platelet and endothelial cell adhesion molecule 1
PVN: Paraventricular nucleus
QUIN: Quinolinic acid
RVLM: Rostral ventrolateral medulla
S100B: S100 calcium-binding protein B
TJs: Tight junctions
TRP: Tryptophan
VCAM-1: Vascular cell adhesion molecule 1

## References

[1] Abbott NJ, Rönnbäck L, Hansson E (2006). Astrocyte-endothelial interactions at the blood-brain barrier. Nat Rev Neurosci..

[2] Zlokovic BV (2008). The blood-brain barrier in health and chronic neurodegenerative disorders. Neuron..

[3] Winkler EA, Bell RD, Zlokovic BV (2011). Central nervous system pericytes in health and disease. Nat Neurosci..

[4] Lo EH, Rosenberg GA (2009). The neurovascular unit in health and disease introduction. Stroke.

[5] Segura I, De Smet F, Hohensinner PJ, de Almodovar CR, Carmeliet P (2009). The neurovascular link in health and disease: an update. Trends Mol Med..

[6] Iadecola C (2010). The overlap between neurodegenerative and vascular factors in the pathogenesis of dementia. Acta Neuropathol..

[7] Daneman R (2012). The blood-brain barrier in health and disease. Ann Neurol..

[8] Hawkins BT (2005). The blood-brain barrier/neurovascular unit in health and disease. Pharmacol Rev..

[9] Reese TS, Karnovsky MJ (1967). Fine structural localization of a blood-brain barrier to exogenous peroxidase. J Cell Biol..

[10] Nabeshima S, Reese TS, Landis DMD, Brightman MW (1975). Junctions in the meninges and marginal glia. J Comp Neurol..

[11] Naghavi M, Abajobir AA, Abbafati C, Abbas KM, Abd-Allah F, Abera SF (2017). Global, regional, and national age-sex specific mortality for 264 causes of death, 1980–2016: a systematic analysis for the Global Burden of Disease Study 2016. Lancet..

[12] Møllgård K, Saunders NR (1986). The development of the human blood-brain and blood-CSF barriers. Neuropathol Appl Neurobiol..

[13] Rascher G, Wolburg H (1997). The tight junctions of the leptomeningeal blood-cerebrospinal fluid barrier during development. J brain Res..

[14] Medwell J, Wray D (2007). Handwriting: what do we know and what do we need to know?. Literacy..

[15] Kniesel U, Wolburg H (2000). Tight junctions of the blood-brain barrier. Cell Mol Neurobiol..

[16] Bell RD, Winkler EA, Sagare AP, Singh I, LaRue B, Deane R (2010). pericytes control key neurovascular functions and neuronal phenotype in the adult brain and during brain aging. Neuron..

[17] Shlosberg D, Benifla M, Kaufer D, Friedman A. Blood-brain barrier breakdown as a therapeutic target in traumatic brain injury. Nat Rev Neurol. 2010;6:393–403. 10.1038/nrneurol.2010.74. 10.1038/nrneurol.2010.74 PMC3625732 20551947

[18] Rapoport B, Adams RJ (1976). Induction of refractoriness to thyrotropin stimulation in cultured thyroid cells. Dependence on new protein synthesis. J Biol Chem..

[19] Grimm A, Friedland K, Eckert A (2016). Mitochondrial dysfunction: the missing link between aging and sporadic Alzheimer’s disease. Biogerontology..

[20] Cani PD, Bibiloni R, Knauf C, Neyrinck AM, Delzenne NM (2008). Changes in gut microbiota control metabolic diet–induced obesity and diabetes in mice. Diabetes..

[21] León-Pedroza JI, González-Tapia LA, del Olmo-Gil E, Castellanos-Rodríguez D, Escobedo G, González-Chávez A (2015). Low-grade systemic inflammation and the development of metabolic diseases: From the molecular evidence to the clinical practice. Cirugía y Cir.

[22] Flegal KM, Kruszon-Moran D, Carroll MD, Fryar CD, Ogden CL (2016). Trends in obesity among adults in the United States, 2005 to 2014. JAMA.

[23] Baeten KM, Akassoglou K (2011). Extracellular matrix and matrix receptors in blood-brain barrier formation and stroke. Dev Neurobiol..

[24] Kelleher RJ, Soiza RL (2013). Evidence of endothelial dysfunction in the development of Alzheimer’s disease: Is Alzheimer’s a vascular disorder?. Am J Cardiovasc Dis..

[25] Kim SY, Buckwalter M, Soreq H, Vezzani A, Kaufer D (2012). Blood-brain barrier dysfunction-induced inflammatory signaling in brain pathology and epileptogenesis. Epilepsia..

[26] Van Vliet EA, Araújo SDC, Redeker S, Van Schaik R, Aronica E, Gorter JA (2007). Blood-brain barrier leakage may lead to progression of temporal lobe epilepsy. Brain..

[27] Abbott NJ, Patabendige AAK, Dolman DEM, Yusof SR, Begley DJ (2010). Structure and function of the blood-brain barrier. Neurobiol Dis..

[28] Marchi N, Granata T, Alexopoulos A, Janigro D (2012). The blood-brain barrier hypothesis in drug resistant epilepsy. Brain..

[29] Raabe A, Schmitz AK, Pernhorst K, Grote A, Von Der Brelie C, Urbach H (2012). Cliniconeuropathologic correlations show astroglial albumin storage as a common factor in epileptogenic vascular lesions. Epilepsia..

[30] Réus GZ, Fries GR, Stertz L, Badawy M, Passos IC, Barichello T (2015). The role of inflammation and microglial activation in the pathophysiology of psychiatric disorders. Neuroscience..

[31] Allan SM, Rothwell NJ (2001). Cytokines and acute neurodegeneration. Nat Rev Neurosci..

[32] Hassan W, Noreen H, Castro-Gomes V, Mohammadzai I, Batista Teixeira da Rocha J, Landeira-Fernandez J. Association of oxidative stress with psychiatric disorders. Curr Pharm Des. 2016;22:2960–2974. doi:10.2174/1381612822666160307145931. 10.2174/1381612822666160307145931 26951103

[33] Sen S, Duman R, Sanacora G (2008). Serum brain-derived neurotrophic factor, depression, and Antidepressant Medications: Meta-Analyses And Implications. Biol Psychiatry..

[34] Kronfol Z, Remick DG (2000). Cytokines and the brain: implications for clinical psychiatry. Am J Psychiatry..

[35] Pollak TA, Drndarski S, Stone JM, David AS, McGuire P, Abbott NJ (2018). The blood–brain barrier in psychosis. Lancet Psychiatry..

[36] Booth FW, Laye MJ (2009). Lack of adequate appreciation of physical exercise’s complexities can pre-empt appropriate design and interpretation in scientific discovery. J Physiol..

[37] Szalewska D, Radkowski M, Demkow U, Winklewski PJ (2017). Exercise strategies to counteract brain aging effects. Adv Exp Med Biol..

[38] Stehouwer CD, Ferreira I, Safar ME, O’Rourke MF (2006). Diabetes, lipids and other cardiovascular risk factors. Arterial stiffness in Hypertension.

[39] Hotamisligil GS. Inflammation and metabolic disorders. Nature. 2006;444:860–7. 10.1038/nature05485. 10.1038/nature05485 17167474

[40] Wellen KE, Hotamisligil GS (2005). Inflammation, stress, and diabetes. J Clin Invest..

[41] Hermann A, Butz S, Stappert J, Weissig H, Kemler R, Hoschuetzky H (1994). Assembly of the cadherin-catenin complex in vitro with recombinant proteins. J Cell Sci..

[42] Wellen KE, Fucho R, Gregor MF, Furuhashi M, Morgan C, Lindstad T (2007). Coordinated regulation of nutrient and inflammatory responses by STAMP2 is essential for metabolic homeostasis. Cell..

[43] Houstis N, Rosen ED, Lander ES (2006). Reactive oxygen species have a causal role in multiple forms of insulin resistance. Nature..

[44] Rajkovic N, Zamaklar M, Lalic K, Jotic A, Lukic L, Milicic T (2014). Relationship between obesity, adipocytokines and inflammatory markers in type 2 diabetes: relevance for cardiovascular risk prevention. Int J Environ Res Public Health..

[45] Reinehr T, Karges B, Meissner T, Wiegand S, Stoffel-Wagner B, Holl RW (2016). Inflammatory markers in obese adolescents with type 2 diabetes and their relationship to hepatokines and adipokines. J Pediatr..

[46] Spranger J, Kroke A, Möhlig M, Hoffmann K, Bergmann MM, Ristow M (2003). Inflammatory cytokines and the risk to develop type 2 diabetes: results of the prospective population-based European Prospective Investigation into Cancer and Nutrition (EPIC)-Potsdam study. Diabetes..

[47] Gironès X, Cruz-Sánchez CZ, Ortega A, Sasaki N, Makita Z, Lafuente JV, Kalaria R, Cruz-Sánchez FF (2004). Nϵ-Carboxymethyllysine in brain aging, diabetes mellitus, and Alzheimer’s disease. Free Radic Biol Med.

[48] Takeda S, Sato N, Ikimura K, Nishino H, Rakugi H, Morishita R (2013). Increased blood-brain barrier vulnerability to systemic inflammation in an Alzheimer disease mouse model. Neurobiol Aging..

[49] Holland WL, Knotts TA, Chavez JA, Wang L-P, Hoehn KL, Summers SA (2007). Lipid mediators of insulin resistance. Nutr Rev..

[50] De La Monte SM (2012). Triangulated mal-signaling in Alzheimer’s disease: Roles of neurotoxic ceramides, ER stress, and insulin resistance reviewed. J Alzheimer’s Dis..

[51] Dubé JJ, Amati F, Toledo FGS, Stefanovic-Racic M, Rossi A, Coen P (2011). Effects of weight loss and exercise on insulin resistance, and intramyocellular triacylglycerol, diacylglycerol and ceramide. Diabetologia..

[52] Kasumov T, Solomon TPJ, Hwang C, Huang H, Haus JM, Zhang R (2015). Improved insulin sensitivity after exercise training is linked to reduced plasma C14:0 ceramide in obesity and type 2 diabetes. Obesity..

[53] Abd El-Kader SM (2011). Aerobic versus resistance exercise training in modulation of insulin resistance, adipocytokines and inflammatory cytokine levels in obese type 2 diabetic patients. J Adv Res..

[54] De Senna PN, Xavier LL, Bagatini PB, Saur L, Galland F, Zanotto C (2015). Physical training improves non-spatial memory, locomotor skills and the blood brain barrier in diabetic rats. Brain Res..

[55] Esposito K, Pontillo A, Di Palo C, Giugliano G, Masella M, Marfella R (2003). Effect of weight loss and lifestyle changes on vascular inflammatory markers in obese women: a randomized trial. J Am Med Assoc..

[56] Goldhammer E, Tanchilevitch A, Maor I, Beniamini Y, Rosenschein U, Sagiv M (2005). Exercise training modulates cytokines activity in coronary heart disease patients. Int J Cardiol..

[57] Taaffe DR, Harris TB, Ferrucci L, Rowe J, Seeman TE (2000). Cross-sectional and prospective relationships of interleukin-6 and c-reactive protein with physical performance in elderly persons: MacArthur studies of successful aging. J Gerontol A Biol Sci Med Sci..

[58] Trøseid M, Lappegård KT, Claudi T, Damås JK, Mørkrid L, Brendberg R (2004). Exercise reduces plasma levels of the chemokines MCP-1 and IL-8 in subjects with the metabolic syndrome. Eur Heart J..

[59] Marcell TJ, McAuley KA, Traustadóttir T, Reaven PD (2005). Exercise training is not associated with improved levels of C-reactive protein or adiponectin. Metabolism..

[60] Pedersen BK (2011). Muscles and their myokines. J Exp Biol..

[61] Petersen AMW (2005). The anti-inflammatory effect of exercise. J Appl Physiol..

[62] Prokopchuk O, Liu Y, Wang L, Wirth K, Schmidtbleicher D, Steinacker JM (2007). Skeletal muscle IL-4, IL-4Rα, IL-13 and IL-13Rα1 expression and response to strength training. Exerc Immunol Rev..

[63] Pedersen BK, Fischer CP (2007). Physiological roles of muscle-derived interleukin-6 in response to exercise. Curr Opin Clin Nutr Metab Care..

[64] Handschin C, Spiegelman BM (2008). The role of exercise and PGC1α in inflammation and chronic disease. Nature..

[65] Nishida Y, Iyadomi M, Higaki Y, Tanaka H, Kondo Y, Otsubo H (2015). Association between the PPARGC1A polymorphism and aerobic capacity in Japanese middle-aged men. Intern Med..

[66] Aronson D, Sheikh-Ahmad M, Avizohar O, Kerner A, Sella R, Bartha P (2004). C-Reactive protein is inversely related to physical fitness in middle-aged subjects. Atherosclerosis..

[67] Chupel MU, Minuzzi LG, Furtado GE, Santos ML, Hogervorst E, Filaire E (2018). Exercise and taurine in inflammation, cognition, and peripheral markers of blood-brain barrier integrity in older women. Appl Physiol Nutr Metab.

[68] Stone TW, Forrest CM, Mackay GM, Stoy N, Darlington LG (2007). Tryptophan, adenosine, neurodegeneration and neuroprotection. Metab Brain Dis..

[69] Ball HJ, Sanchez-Perez A, Weiser S, Austin CJD, Astelbauer F, Miu J (2007). Characterization of an indoleamine 2,3-dioxygenase-like protein found in humans and mice. Gene..

[70] Takao S, Sumisugu N, Hirata F, Hayaishi O (1978). Indoleamine 2,3-dioxygenase. Purification and some properties. J Biol Chem..

[71] Stone TW (2007). Kynurenic acid blocks nicotinic synaptic transmission to hippocampal interneurons in young rats. Eur J Neurosci..

[72] Sas K, Robotka H, Toldi J, Vécsei L (2007). Mitochondria, metabolic disturbances, oxidative stress and the kynurenine system, with focus on neurodegenerative disorders. J Neurol Sci..

[73] Guillemin GJ (2012). Quinolinic acid, the inescapable neurotoxin. FEBS J..

[74] Cervenka I, Agudelo LZ, Ruas JL (2017). Kynurenines: Tryptophan’s metabolites in exercise, inflammation, and mental health. Science.

[75] Guillemin GJ, Kerr SJ, Smythe GA, Smith DG, Kapoor V, Armati PJ (2001). Kynurenine pathway metabolism in human astrocytes: a paradox for neuronal protection. J Neurochem..

[76] Werner-Felmayer G, Werner ER, Fuchs D, Hausen A, Reibnegger G, Wachter H (1989). Characteristics of interferon induced tryptophan metabolism in human cells in vitro. BBA - Mol Cell Res..

[77] Schlittler M, Goiny M, Agudelo LZ, Venckunas T, Brazaitis M, Skurvydas A (2016). Endurance exercise increases skeletal muscle kynurenine aminotransferases and plasma kynurenic acid in humans. Am J Physiol Cell Physiol..

[78] András IE, Deli MA, Veszelka S, Hayashi K, Hennig B, Toborek M (2007). The NMDA and AMPA/KA receptors are involved in glutamate-induced alterations of occludin expression and phosphorylation in brain endothelial cells. J Cereb Blood Flow Metab..

[79] Beggiato S, Antonelli T, Tomasini MC, Tanganelli S, Fuxe K, Schwarcz R (2013). Kynurenic acid, by targeting α7 nicotinic acetylcholine receptors, modulates extracellular GABA levels in the rat striatum in vivo. Eur J Neurosci..

[80] Hilmas C, Pereira EFR, Alkondon M, Rassoulpour A, Schwarcz R, Albuquerque EX (2001). The brain metabolite kynurenic acid inhibits alpha7 nicotinic receptor activity and increases non-alpha7 nicotinic receptor expression: physiopathological implications. J Neurosci..

[81] Konradsson-Geuken Å, Wu HQ, Gash CR, Alexander KS, Campbell A, Sozeri Y (2010). Cortical kynurenic acid bi-directionally modulates prefrontal glutamate levels as assessed by microdialysis and rapid electrochemistry. Neuroscience..

[82] Winklewski PJ, Radkowski M, Wszedybyl-Winklewska M, Demkow U (2015). Brain inflammation and hypertension: the chicken or the egg?. Neuroinflammation..

[83] Winklewski PJ, Radkowski M, Demkow U (2016). Neuroinflammatory mechanisms of hypertension: potential therapeutic implications. Curr Opin Nephrol Hypertens..

[84] Biancardi VC, Stern JE (2016). Compromised blood-brain barrier permeability: novel mechanism by which circulating angiotensin II signals to sympathoexcitatory centres during hypertension. J Physiol..

[85] Carey RM, Wang ZQ, Siragy HM (2000). Role of the angiotensin type 2 receptor in the regulation of blood pressure and renal function. Hypertension..

[86] Suzuki Y, Ruiz-Ortega M, Lorenzo O, Ruperez M, Esteban V, Egido J (2003). Inflammation and angiotensin II. Int J Biochem Cell Biol..

[87] Muller DN, Shagdarsuren E, Park JK, Dechend R, Mervaala E, Hampich F (2002). Immunosuppressive treatment protects against angiotensin II-induced renal damage. Am J Pathol..

[88] Shi P, Diez-Freire C, Jun JY, Qi Y, Katovich MJ, Li Q (2010). Brain microglial cytokines in neurogenic hypertension. Hypertension..

[89] Waki H, Gouraud SS, Maeda M, Raizada MK, Paton JFR (2011). Contributions of vascular inflammation in the brainstem for neurogenic hypertension. Respir Physiol Neurobiol..

[90] Zubcevic J, Waki H, Raizada M, Paton J (2011). Autonomic-immune-vascular dysfunction: an emerging concept for neurogenic hypertension. Hypertension..

[91] Ito H, Takemori K, Kawai J, Suzuki T (2000). AT1 receptor antagonist prevents brain edema without lowering blood pressure. Brain Edema XI..

[92] de Vries HE, Kuiper J, de Boer AG, Van Berkel TJC, Breimer DD (1997). The blood-brain barrier in neuroinflammatory diseases. Pharmacol Rev..

[93] Zhang M, Mao Y, Ramirez SH, Tuma RF, Chabrashvili T (2010). Angiotensin II induced cerebral microvascular inflammation and increased blood-brain barrier permeability via oxidative stress. Neuroscience..

[94] Pan YX, Gao L, Wang WZ, Zheng H, Liu D, Patel KP (2007). Exercise training prevents arterial baroreflex dysfunction in rats treated with central angiotensin II. Hypertension..

[95] Agarwal D, Welsch MA, Keller JN, Francis J (2011). Chronic exercise modulates RAS components and improves balance between pro-and anti-inflammatory cytokines in the brain of SHR. Basic Res Cardiol..

[96] Chaar LJ, Alves TP, Junior AMB, Michelini LC (2015). Early training-induced reduction of angiotensinogen in autonomic areas-the main effect of exercise on brain renin-angiotensin system in hypertensive rats. PLoS One..

[97] Jia LL, Kang YM, Wang FX, Li HB, Zhang Y, Yu XJ, et al. Exercise training attenuates hypertension and cardiac hypertrophy by modulating neurotransmitters and cytokines in hypothalamic paraventricular nucleus. PLoS One. 2014;9 10.1371/journal.pone.0085481. 10.1371/journal.pone.0085481 PMC3901693 24482680

[98] Negrão CE, Moreira ED, Brum PC, Denadai ML, Krieger EM (1992). Vagal and sympathetic control of heart rate during exercise by sedentary and exercise-trained rats. Braz J Med Biol Res..

[99] Sugawara J, Murakami H, Maeda S, Kuno S, Matsuda M (2001). Change in post-exercise vagal reactivation with exercise training and detraining in young men. Eur J Appl Physiol..

[100] Buttler L, Jordão MT, Fragas MG, Ruggeri A, Ceroni A, Michelini LC (2017). Maintenance of blood-brain barrier integrity in hypertension: a novel benefit of exercise training for autonomic control. Front Physiol.

[101] Meshorer E (2005). Chronic cholinergic imbalances promote brain diffusion and transport abnormalities. FASEB J..

[102] Nishihara M, Hirooka Y, Matsukawa R, Kishi T, Sunagawa K (2012). Oxidative stress in the rostral ventrolateral medulla modulates excitatory and inhibitory inputs in spontaneously hypertensive rats. J Hypertens..

[103] Muratani H, Averill DB, Ferrario CM (1991). Effect of angiotensin II in ventrolateral medulla of spontaneously hypertensive rats. Am J Physiol..

[104] Vieira AA, Colombari E, De Luca LA, Colombari DSA, De Paula PM, Menani JV (2010). Importance of angiotensinergic mechanisms for the pressor response to l-glutamate into the rostral ventrolateral medulla. Brain Res..

[105] Colombari E, Sato MA, Cravo SL, Bergamaschi CT, Campos RR, Lopes OU (2001). Role of the medulla oblongata in hypertension. Hypertension..

[106] Kishi T, Hirooka Y, Sunagawa K (2012). Sympathoinhibition caused by orally administered telmisartan through inhibition of the at 1 receptor in the rostral ventrolateral medulla of hypertensive rats. Hypertens Res..

[107] Mills E, Minson J, Drolet G, Chalmers J (1990). Effect of intrathecal amino acid receptor antagonists on basal blood pressure and pressor responses to brainstem stimulation in normotensive and hypertensive rats. J Cardiovasc Pharmacol..

[108] Schreurs J, Seelig T, Schulman H (1986). β2-adrenergic receptors on peripheral nerves. J Neurochem..

[109] Braun V, Clarke V. What can “thematic analysis” offer health and wellbeing researchers? Int J Qual Stud Health Well-being. 2014; 10.3402/qhw.v9.26152. 10.3402/qhw.v9.26152 PMC4201665 25326092

[110] Atzori M, Cuevas-Olguin R, Esquivel-Rendon E, Garcia-Oscos F, Salgado-Delgado RC, Saderi N (2016). Locus ceruleus norepinephrine release: a central regulator of cns spatio-temporal activation?. Front Synaptic Neurosci.

[111] Chandler DJ. Evidence for a specialized role of the locus coeruleus noradrenergic system in cortical circuitries and behavioral operations. Brain Res. 2016;1641 Pt B:197–206. doi:10.1016/j.brainres.2015.11.022. 10.1016/j.brainres.2015.11.022 PMC4879003 26607255

[112] Feinstein DL, Kalinin S, Braun D (2016). Causes, consequences, and cures for neuroinflammation mediated via the locus coeruleus: noradrenergic signaling system. J Neurochem..

[113] O’Donnell J, Zeppenfeld D, McConnell E, Pena S, Nedergaard M (2012). Norepinephrine: a neuromodulator that boosts the function of multiple cell types to optimize CNS performance. Neurochem Res..

[114] Hetier E, Ayala J, Bousseau A, Prochiantz A (1991). Modulation of interleukin-1 and tumor necrosis factor expression by β-adrenergic agonists in mouse ameboid microglial cells. Exp Brain Res..

[115] Frohman EM, Vayuvegula B, van den Noort S, Gupta S (1988). Norepinephrine inhibits gamma-interferon-induced MHC class II (Ia) antigen expression on cultured brain astrocytes. J Neuroimmunol..

[116] Sanders VM (2012). The beta2-adrenergic receptor on T and B lymphocytes: do we understand it yet?. Brain Behav Immun..

[117] Huang HW, Fang XX, Wang XQ, Peng YP, Qiu YH (2014). Regulation of differentiation and function of helper T cells by lymphocyte-derived catecholamines via α1- and β2-adrenoceptors. Neuroimmunomodulation..

[118] Jurič DM, Lončar D, Čarman-Kržan M (2008). Noradrenergic stimulation of BDNF synthesis in astrocytes: mediation via α1- and β1/β2-adrenergic receptors. Neurochem Int..

[119] Middlemas D (2011). Brain derived neurotrophic factor. xPharm Compr Pharmacol Ref.

[120] Zafra F, Lindholm D, Castrén E, Hartikka J, Thoenen H (1992). Regulation of brain-derived neurotrophic factor and nerve growth factor mRNA in primary cultures of hippocampal neurons and astrocytes. J Neurosci..

[121] Schurr A, West CA, Rigor BM (1988). Lactate-supported synaptic function in the rat hippocampal slice preparation. Sci Sci..

[122] Van Hall G, Strømstad M, Rasmussen P, Jans Ø, Zaar M, Gam C (2009). Blood lactate is an important energy source for the human brain. J Cereb Blood Flow Metab..

[123] Benarroch EE (2010). Glycogen metabolism: Metabolic coupling between astrocytes and neurons. Neurology..

[124] Fillenz M, Lowry JP, Boutelle MG, Fray AE (1999). The role of astrocytes and noradrenaline in neuronal glucose metabolism. Acta Physiol Scand..

[125] Hertz L, Lovatt D, Goldman SA, Nedergaard M (2010). Adrenoceptors in brain: cellular gene expression and effects on astrocytic metabolism and [Ca2+]i. Neurochem Int..

[126] Pellerin L, Bouzier-Sore AK, Aubert A, Serres S, Merle M, Costalat R (2007). Activity-dependent regulation of energy metabolism by astrocytes: an update. Glia..

[127] Lewis GD, Farrell L, Wood MJ, Martinovic M, Arany Z, Rowe GC (2010). Metabolic signatures of exercise in human plasma. Sci Transl Med.

[128] Delezie J, Handschin C (2018). Endocrine crosstalk between skeletal muscle and the brain. Front Neurol.

[129] Pellerin L, Pellegri G, Bittar PG, Charnay Y, Bouras C, Martin JL (1998). Evidence supporting the existence of an activity-dependent astrocyte-neuron lactate shuttle. Dev Neurosci..

[130] Bergersen L, Rafiki A, Ottersen OP (2002). Immunogold cytochemistry identifies specialized membrane domains for monocarboxylate transport in the central nervous system. Neurochem Res..

[131] Bergersen LH (2015). Lactate transport and signaling in the brain: potential therapeutic targets and roles in body-brain interaction. J Cereb Blood Flow Metab..

[132] Barros LF (2013). Metabolic signaling by lactate in the brain. Trends Neurosci..

[133] Ruan GX, Kazlauskas A (2013). Lactate engages receptor tyrosine kinases Axl, Tie2, and vascular endothelial growth factor receptor 2 to activate phosphoinositide 3-kinase/AKT and promote angiogenesis. J Biol Chem..

[134] Morland C, Andersson KA, Haugen ØP, Hadzic A, Kleppa L, Gille A (2017). Exercise induces cerebral VEGF and angiogenesis via the lactate receptor HCAR1. Nat Commun..

[135] Todd RD, Botteron KN (2001). Is attention-deficit/hyperactivity disorder an energy deficiency syndrome?. Biol Psychiatry..

[136] Russell VA, Oades RD, Tannock R, Killeen PR, Auerbach JG, Johansen EB (2006). Response variability in attention-deficit/hyperactivity disorder: a neuronal and glial energetics hypothesis. Behav Brain Funct..

[137] Medin T, Medin H, Brandsar Hefte M, Storm-Mathisen J, Bergersen LH (2018). Upregulation of the lactate transporter monocarboxylate transporter 1 at the blood-brain barrier in a rat model of attention-deficit/hyperactivity disorder suggests hyperactivity could be a form of self-treatment. Behav Brain Res..

[138] Dalgas U, Stenager E (2012). Exercise and disease progression in multiple sclerosis: can exercise slow down the progression of multiple sclerosis?. Ther Adv Neurol Disord..

[139] Miller DH, Khan OA, Sheremata WA, Blumhardt LD, Rice GPA, Libonati MA (2003). A controlled trial of natalizumab for relapsing multiple sclerosis. N Engl J Med..

[140] Paterson P (1976). Experimental allergic encephalomyelitis: role of fibrin deposition in immunopathogenesis of inflammation in rats. Fed Proc..

[141] Mokhtarzade M, Motl R, Negaresh R, Zimmer P, Khodadoost M, Baker JS (2018). Exercise-induced changes in neurotrophic factors and markers of blood-brain barrier permeability are moderated by weight status in multiple sclerosis. Neuropeptides.

[142] White LJ, Castellano V (2008). Exercise and brain health: Implications for multiple sclerosis: Part II immune factors and stress hormones. Sport Med..

[143] Rossi S, Furlan R, De Chiara V, Musella A, Lo Giudice T, Mataluni G (2009). Exercise attenuates the clinical, synaptic and dendritic abnormalities of experimental autoimmune encephalomyelitis. Neurobiol Dis..

[144] van Praag H (2005). Exercise enhances learning and hippocampal neurogenesis in aged mice. J Neurosci..

[145] Cotman CW, Berchtold NC, Christie LA (2007). Exercise builds brain health: key roles of growth factor cascades and inflammation. Trends Neurosci..

[146] Castellano V, White LJ (2008). Serum brain-derived neurotrophic factor response to aerobic exercise in multiple sclerosis. J Neurol Sci..

[147] Leavitt VM, Cirnigliaro C, Cohen A, Farag A, Brooks M, Wecht JM (2014). Aerobic exercise increases hippocampal volume and improves memory in multiple sclerosis: preliminary findings. Neurocase..

[148] Motl RW, Pilutti LA, Learmonth YC, Goldman MD, Brown T (2013). Clinical importance of steps taken per day among persons with multiple sclerosis. PLoS One..

[149] Beier M, Bombardier CH, Hartoonian N, Motl RW, Kraft GH (2014). Improved physical fitness correlates with improved cognition in multiple sclerosis. Arch Phys Med Rehabil..

[150] Prakash RS, Snook EM, Erickson KI, Colcombe SJ, Voss MW, Motl RW (2007). Cardiorespiratory fitness: a predictor of cortical plasticity in multiple sclerosis. Neuroimage..

[151] Prakash RS, Snook EM, Motl RW, Kramer AF (2010). Aerobic fitness is associated with gray matter volume and white matter integrity in multiple sclerosis. Brain Res..

[152] Gamba P, Testa G, Gargiulo S, Staurenghi E, Poli G, Leonarduzzi G (2015). Oxidized cholesterol as the driving force behind the development of Alzheimer’s disease. Front Aging Neurosci.

[153] Mullins RJ, Diehl TC, Chia CW, Kapogiannis D (2017). Insulin resistance as a link between amyloid-beta and tau pathologies in Alzheimer’s disease. Front Aging Neurosci.

[154] Bednarczyk J, Lukasiuk K (2011). Tight junctions in neurological diseases. Acta Neurobiol Exp..

[155] Gonçalves A, Ambrósio AF, Fernandes R (2013). Regulation of claudins in blood-tissue barriers under physiological and pathological states. Tissue Barriers..

[156] Krabbe G, Halle A, Matyash V, Rinnenthal JL, Eom GD, Bernhardt U (2013). Functional impairment of microglia coincides with beta-amyloid deposition in mice with Alzheimer-like pathology. PLoS One..

[157] Lepelletier FX, Mann DMA, Robinson AC, Pinteaux E, Boutin H (2017). Early changes in extracellular matrix in Alzheimer’s disease. Neuropathol Appl Neurobiol..

[158] Holmes C (2017). Inflammation in Alzheimer’s disease. Dementia, Fifth Ed..

[159] He X, Liu D, Zhang Q, Liang F, Dai G, Zeng J (2017). Voluntary exercise promotes glymphatic clearance of amyloid beta and reduces the activation of astrocytes and microglia in aged mice. Front Mol Neurosci..

[160] Jensen CS, Hasselbalch SG, Waldemar G, Simonsen AH (2015). Biochemical markers of physical exercise on mild cognitive impairment and dementia: Systematic review and perspectives. Front Neurol.

[161] Baker LD, Frank LL, Foster-Schubert K, Green PS, Wilkinson CW, McTiernan A (2010). Effects of aerobic exercise on mild cognitive impairment: a controlled trial. Arch Neurol..

[162] Voss MW, Vivar C, Kramer AF, van Praag H (2013). Bridging animal and human models of exercise-induced brain plasticity. Trends Cogn Sci..

[163] Smith PJ, Blumenthal JA, Hoffman BM, Cooper H, Strauman TA, Welsh-Bohmer K (2010). Aerobic exercise and neurocognitive performance: a meta-analytic review of randomized controlled trials. Psychosom Med..

[164] Groot C, Hooghiemstra AM, Raijmakers PGHM, van Berckel BNM, Scheltens P, Scherder EJA (2016). The effect of physical activity on cognitive function in patients with dementia: a meta-analysis of randomized control trials. Ageing Res Rev..

[165] Öhman H, Savikko N, Strandberg TE, Pitkälä KH (2014). Effect of physical exercise on cognitive performance in older adults with mild cognitive impairment or dementia: a systematic review. Dement Geriatr Cogn Disord..

[166] Nieman DC (2003). Current perspective on exercise immunology. Curr Sports Med Rep..

[167] Gleeson M (2007). Immune function in sport and exercise. J Appl Physiol..

[168] Roh H-T, Cho S-Y, Yoon H-G, So W-Y (2017). Effect of exercise intensity on neurotrophic factors and blood–brain barrier permeability induced by oxidative–nitrosative stress in male college students. Int J Sport Nutr Exerc Metab..

[169] Kasapis C, PD T. The effects of physical activity on serum C-reactive protein and inflammatory markers: a systematic review. J Am Coll Cardiol. 2005;45:1563–9. 10.1016/j.jacc.2004.12.077. 10.1016/j.jacc.2004.12.077 15893167

[170] Plaisance EP, Grandjean PW (2006). Physical activity and high-sensitivity C-reactive protein. Sport Med..

[171] Koh SXT, Lee JKW (2014). S100B as a marker for brain damage and blood-brain barrier disruption following exercise. Sport Med..

[172] Marchi N, Cavaglia M, Fazio V, Bhudia S, Hallene K, Janigro D (2004). Peripheral markers of blood-brain barrier damage. Clin Chim Acta..

[173] Roh HT, Cho SY, So WY (2017). Obesity promotes oxidative stress and exacerbates blood-brain barrier disruption after high-intensity exercise. J Sport Heal Sci..

[174] Anderson JM, Van Itallie CM (2009). Physiology and function of the tight junction. Cold Spring Harb Perspect Biol..

[175] Stålnacke BM, Tegner Y, Sojka P (2004). Playing soccer increases serum concentrations of the biochemical markers of brain damage S-100B and neuron-specific enolase in elite players: a pilot study. Brain Inj..

[176] Riuzzi F, Sorci G, Beccafico S, Donato R. S100B engages RAGE or bFGF/FGFR1 in myoblasts depending on its own concentration and myoblast density. implications for muscle regeneration. PLoS One. 2012;7 10.1371/journal.pone.0028700 PMC3262793 22276098

[177] Shanker Sharma H, Cervós-Navarro J, Kumar DP (1991). Increased blood-brain barrier permeability following acute short-term swimming exercise in conscious normotensive young rats. Neurosci Res..

[178] Bailey DM, Evans KA, Mceneny J, Young IS, Hullin DA, James PE (2011). Exercise-induced oxidative-nitrosative stress is associated with impaired dynamic cerebral autoregulation and blood-brain barrier leakage. Exp Physiol..

[179] Görgens SW, Eckardt K, Jensen J, Drevon CA, Eckel J (2015). Exercise and regulation of adipokine and myokine production. Prog Mol Biol Transl Sci..

[180] Gleeson M, McFarlin B, Flynn M (2006). Exercise and toll-like receptors. Exerc Immunol Rev..

[181] Esser N, Legrand-Poels S, Piette J, Scheen AJ, Paquot N (2014). Inflammation as a link between obesity, metabolic syndrome and type 2 diabetes. Diabetes Res Clin Pract..

[182] Kristiansen OP, Mandrup-Poulsen T. Interleukin-6 andand diabetes: the good, the bad, or the indifferent? Diabetes. 2005;54(suppl 2):114–24. 10.2337/diabetes.54.suppl_2.S114. 10.2337/diabetes.54.suppl_2.s114 16306329

[183] Gmiąt A, Jaworska J, Micielska K, Kortas J, Prusik K, Prusik K (2018). Improvement of cognitive functions in response to a regular Nordic walking training in elderly women—a change dependent on the training experience. Exp Gerontol..

[184] Ostrowski K, Rohde T, Asp S, Schjerling P, Pedersen BK (1999). Pro- and anti-inflammatory cytokine balance in strenuous exercise in humans. J Physiol..

[185] Ostrowski K, Schjerling P, Pedersen BK (2000). Physical activity and plasma interleukin-6 in humans—effect of intensity of exercise. Eur J Appl Physiol..

[186] Akira S, Taga T, Kishimoto T (1993). Interleukin-6 in biology and medicine. Adv Immunol..

[187] Ostrowski K, Rohde T, Zacho M, Asp S, Pedersen BK (1998). Evidence that interleukin-6 is produced in human skeletal muscle during prolonged running. J Physiol..

[188] Jonsdottir IH, Schjerling P, Ostrowski K, Asp S, Richter EA. Muscle contractions induce interleukin-6 mRNA production in rat skeletal muscles. J Physiol. 2000;528(Pt 1):157–63. 10.1111/j.1469-7793.2000.00157.x. 10.1111/j.1469-7793.2000.00157.x PMC2270126 11018114

[189] Steensberg A, Van Hall G, Osada T, Sacchetti M, Saltin B, Pedersen BK (2000). Production of interleukin-6 in contracting human skeletal muscles can account for the exercise-induced increase in plasma interleukin-6. J Physiol..

[190] Abramson JL, Vaccarino V (2002). Relationship between physical activity and inflammation among apparently healthy middle-aged and older US adults. Arch Intern Med..

[191] Gomez-Cabrera MC, Domenech E, Viña J (2008). Moderate exercise is an antioxidant: Upregulation of antioxidant genes by training. Free Radic Biol Med..

[192] Ji L (2008). Modulation of skeletal muscle antioxidant defense by exercise: role of redox signaling. Free Radic Biol Med..

[193] Teixeira-Lemos E, Nunes S, Teixeira F, Reis F (2011). Regular physical exercise training assists in preventing type 2 diabetes development: focus on its antioxidant and anti-inflammatory properties. Cardiovasc Diabetol..

[194] McKee AC, Daneshvar DH, Alvarez VE, Stein TD (2014). The neuropathology of sport. Acta Neuropathol..

[195] Nguyen A, Duquette N, Mamarbachi M, Thorin E (2016). Epigenetic regulatory effect of exercise on glutathione peroxidase 1 expression in the skeletal muscle of severely dyslipidemic mice. PLoS One..

[196] Qi Z, He J, Zhang Y, Shao Y, Ding S (2011). Exercise training attenuates oxidative stress and decreases p53 protein content in skeletal muscle of type 2 diabetic Goto-Kakizaki rats. Free Radic Biol Med.

[197] Wolburg H, Lippoldt A (2002). Tight junctions of the blood-brain barrier: development, composition and regulation. Vascul Pharmacol..

[198] Liebner S, Fischmann A, Rascher G, Duffner F, Grote E-H, Kalbacher H (2000). Claudin-1 and claudin-5 expression and tight junction morphology\rare altered in blood vessels of human glioblastoma multiforme. Acta Neuropathol..

[199] Morita K, Sasaki H, Furuse M, Tsukita S (1999). Endothelial claudin: claudin-5/TMVCF constitutes tight junction strands in endothelial cells. J Cell Biol..

[200] Nitta T, Hata M, Gotoh S, Seo Y, Sasaki H, Hashimoto N (2003). Size-selective loosening of the blood-brain barrier in claudin-5-deficient mice. J Cell Biol..

[201] Furuse M, Fujita K, Hiiragi T, Fujimoto K, Tsukita S (1998). Claudin-1 and -2: novel integral membrane proteins localizing at tight junctions with no sequence similarity to occludin. J Cell Biol..

[202] Balda MS, Whitney JA, Flores C. Functional dissociation of paracellular permeability and transepithelial electrical resistance and disruption of the apical-basolateral intramembrane diffusion barrier by expression of a mutant tight junction membrane protein. J Cell Biol. 1996;134:1031–49. 10.1083/jcb.134.4.1031. 10.1083/jcb.134.4.1031 PMC2120963 8769425

[203] Tsukita S, Furuse M, Itoh M. Structural and signalling molecules come together at tight junctions Shoichiro Tsukita *, Mikio Furuse and Masahiko Itoh. Curr Opin Cell Biol. 1999;11:628–33. Figure 1: 10.1016/S0955-0674(99)00016-2. 10.1016/s0955-0674(99)00016-2 10508648

[204] Tsukita S, Itoh M (2001). Multifunctional strands in tight junctions. Nat Rev..

[205] Wong AST, Gumbiner BM (2003). Adhesion-independent mechanism for suppression of tumor cell invasion by E-cadherin. J Cell Biol..

[206] Greenwood J, Amos CL, Walters CE, Couraud P-O, Lyck R, Engelhardt B (2003). Intracellular domain of brain endothelial intercellular adhesion molecule-1 is essential for T lymphocyte-mediated signaling and migration. J Immunol..

[207] Oppenheimer-Marks N, Davis LS, Bogue DT, Ramberg J, Lipsky PE (1991). Differential utilization of ICAM-1 and VCAM-1 during the adhesion and transendothelial migration of human T lymphocytes. J Immunol..

[208] del Zoppo GJ, Milner R (2006). Integrin-Matrix Interactions in the Cerebral Microvasculature. Arterioscler Thromb Vasc Biol..

[209] Alvarez JI, Cayrol R, Prat A (1812). Disruption of central nervous system barriers in multiple sclerosis. Biochim Biophys Acta.

[210] Larochelle C, Alvarez JI, Prat A (2011). How do immune cells overcome the blood-brain barrier in multiple sclerosis?. FEBS Lett..

[211] Weiss N, Miller F, Cazaubon S, Couraud PO (2009). The blood-brain barrier in brain homeostasis and neurological diseases. Biochim Biophys Acta.

[212] Raleigh DR, Boe DM, Yu D, Weber CR, Marchiando AM, Bradford EM (2011). Occludin S408 phosphorylation regulates tight junction protein interactions and barrier function. J Cell Biol..

[213] Jia JP, Meng R, Sun YX, Sun WJ, Ji XM, Jia LF (2005). Cerebrospinal fluid tau, Aβ1-42and inflammatory cytokines in patients with Alzheimer’s disease and vascular dementia. Neurosci Lett..

[214] Wen H, Watry DD, Marcondes MCG, Fox HS (2004). Selective decrease in paracellular conductance of tight junctions: role of the first extracellular domain of claudin-5. Mol Cell Biol..

[215] Souza PS, Gonçalves ED, Pedroso GS, Farias HR, Junqueira SC, Marcon R (2017). Physical exercise attenuates experimental autoimmune encephalomyelitis by inhibiting peripheral immune response and blood-brain barrier disruption. Mol Neurobiol..

[216] Schreibelt G, Musters RJP, Reijerkerk A, de Groot LR, van der Pol SMA, Hendrikx EML, et al. Lipoic acid affects cellular migration into the central nervous system and stabilazes blood-brain barrier integrity. J Immunol. 2006;177:2630–7. 10.4049/jimmunol.177.4.2630. 10.4049/jimmunol.177.4.2630 16888025

[217] Ramirez SH, Fan S, Dykstra H, Rom S, Mercer A, Reichenbach NL, et al. Inhibition of glycogen synthase kinase 3β promotes tight junction stability in brain endothelial cells by half-life extension of occludin and claudin-5. PLoS One. 2013;8. 10.1371/journal.pone.0055972. 10.1371/journal.pone.0055972 PMC3572160 23418486

[218] Isla AG, Vázquez-Cuevas FG, Peña-Ortega F. Exercise prevents amyloid-β-induced hippocampal network disruption by inhibiting GSK3β activation. J Alzheimer’s Dis. 2016;52:333–43. 10.3233/JAD-150352. 10.3233/JAD-150352 27003207

[219] Ramirez SH, Fan S, Zhang M, Papugani A, Reichenbach N, Dykstra H (2010). Inhibition of glycogen synthase kinase 3β (GSK3β) decreases inflammatory responses in brain endothelial cells. Am J Pathol..

[220] Booth FW, Roberts CK, Laye MJ (2012). Lack of exercise is a major cause of chronic diseases.

[221] Lange-Asschenfeldt C, Kojda G (2008). Alzheimer’s disease, cerebrovascular dysfunction and the benefits of exercise: From vessels to neurons. Exp Gerontol..

[222] Intlekofer KA, Cotman CW (2013). Exercise counteracts declining hippocampal function in aging and Alzheimer’s disease. Neurobiol Dis..

[223] Phillips C, Akif Baktir M, Das D, Lin B, Salehi A (2015). The link between physical activity and cognitive dysfunction in Alzheimer disease. Phys Ther..

[224] Bherer L, Erickson KI, Liu-Ambrose T (2013). A review of the effects of physical activity and exercise on cognitive and brain functions in older adults. J Aging Res..

[225] Muscari A, Giannoni C, Pierpaoli L, Berzigotti A, Maietta P, Foschi E (2010). Chronic endurance exercise training prevents aging-related cognitive decline in healthy older adults: a randomized controlled trial. Int J Geriatr Psychiatry..

[226] Ten Brinke LF, Bolandzadeh N, Nagamatsu LS, Hsu CL, Davis JC, Miran-Khan K (2015). Aerobic exercise increases hippocampal volume in older women with probable mild cognitive impairment: a 6-month randomised controlled trial. Br J Sports Med..

[227] Forbes SC, Forbes D, Forbes S, Blake CM, Chong LY, Thiessen EJ, et al. Exercise interventions for preventing dementia or delaying cognitive decline in people with mild cognitive impairment. Cochrane Database Syst Rev. 2015;2015 10.1002/14651858.CD011706.

[228] Colcombe SJ, Erickson KI, Scalf PE, Kim JS, Prakash R, McAuley E (2006). Aerobic exercise training increases brain volume in aging humans. J Gerontol A Biol Sci Med Sci..

[229] Shukla SK, Kumar S, Selvaraj P, Subba RV (2014). Computerized maintenance management system for indigenously developed fighter aircraft inline with emerging trends. ARPN J Eng Appl Sci..

[230] Tu RH, Zeng ZY, Zhong GQ, Wu WF, Lu YJ, Bo ZD (2014). Effects of exercise training on depression in patients with heart failure: a systematic review and meta-analysis of randomized controlled trials. Eur J Heart Fail..

[231] Rosenbaum S, Tiedemann A, Sherrington C, Curtis J, Ward PB (2014). Physical activity interventions for people with mental illness: a systematic review and meta-analysis. J Clin Psychiatry..

[232] Firth J, Cotter J, Elliott R, French P, Yung AR (2015). A systematic review and meta-analysis of exercise interventions in schizophrenia patients. Psychol Med..

[233] Dauwan M, Begemann MJH, Heringa SM, Sommer IE (2016). Exercise improves clinical symptoms, quality of life, global functioning, and depression in schizophrenia: a systematic review and meta-analysis. Schizophr Bull..

[234] Niebauer J, Maxwell AJ, Lin PS, Tsao PS, Kosek J, Bernstein D (1999). Impaired aerobic capacity in hypercholesterolemic mice: partial reversal by exercise training. Am J Physiol..

[235] Niebauer J, Maxwell AJ, Lin PS, Wang D, Tsao PS, Cooke JP (2003). NOS inhibition accelerates atherogenesis: reversal by exercise. Am J Physiol Hear Circ Physiol..

[236] Adams V, Niebauer J (2015). Reversing heart failure-associated pathophysiology with exercise: what actually improves and by how much?. Heart Fail Clin..

[237] Lenk K, Schuler G, Adams V (2010). Skeletal muscle wasting in cachexia and sarcopenia: molecular pathophysiology and impact of exercise training. J Cachexia Sarcopenia Muscle..

[238] Wilson MG, Ellison GM, Cable NT (2016). Basic science behind the cardiovascular benefits of exercise. Br J Sports Med..

[239] Ziemann E, Zembroń-Lacny A, Kasperska A, Antosiewicz J, Grzywacz T, Garsztka T (2013). Exercise training-induced changes in inflammatory mediators and heat shock proteins in young tennis players. J Sport Sci Med..

[240] Ribeiro F, Alves AJ, Teixeira M, Miranda F, Azevedo C, Duarte JA (2012). Exercise training increases interleukin-10 after an acute myocardial infarction: a randomised clinical trial. Int J Sport Med.

[241] Lin R, Chen F, Wen S, Teng T, Pan Y, Huang H (2018). Interleukin-10 attenuates impairment of the blood-brain barrier in a severe acute pancreatitis rat model. J Inflamm.

[242] Eyre HA, Papps E, Baune BT (2013). Treating depression and depression-like behavior with physical activity: An immune perspective. Front Psychiatry.

[243] Flynn MG, McFarlin BK, Phillips MD, Stewart LK, Timmerman KL (2003). Toll-like receptor 4 and CD14 mRNA expression are lower in resistive exercise-trained elderly women. J Appl Physiol..

[244] McFarlin BK, Flynn MG, Campbell WW, Stewart LK, Timmerman KL. TLR4 is lower in resistance-trained older women and related to inflammatory cytokines. Med Sci Sports Exerc. 2005;37:1876–83. 10.1249/01.MSS.0000145465.71269.10. 10.1249/01.mss.0000145465.71269.10 15514501

[245] McFarlin BK, Flynn MG, Campbell WW, Craig BA, Robinson JP, Stewart LK (2006). Physical activity status, but not age, influences inflammatory biomarkers and toll-like receptor 4. J GerontolA Biol Sci Med Sci..

[246] Banchereau J, Steinman RM. Dendritic cells and the control of immunology. 1998. doi:10.1038/32588. 10.1038/32588 9521319

[247] Pasare C, Medzhitov R (2004). Toll-like receptors: linking innate and adaptive immunity. Mech Lymph Act Immune Regul X..

[248] Takeda K, Akira S (2004). TLR signaling pathways. Semin Immunol..

[249] National Institute of Clinical Excellence (2009). Depression in adults: the treatment and management of depression in adults | depression | Information for the public | NICE.

[250] WHO (2015). Fiscal policies for diet and the prevention of noncommunicable diseases.

[251] Raja R, Rosenberg GA, Caprihan A (2018). MRI measurements of blood-brain barrier function in dementia: a review of recent studies. Neuropharmacol.

